# Affective wellbeing moderates the association between polygenic risk score for neuroticism and change in neuroticism

**DOI:** 10.1192/j.eurpsy.2023.422

**Published:** 2023-07-19

**Authors:** J. Bahbouhová, M. V. Cade, A. T. De Sadeleer, C. Dibbets, L.-Q. Herrmann, P. O. F. Hovens, B. M. Jakson, R. C. Reising, C. Menne-Lothmann, J. Decoster, R. van Winkel, D. Collip, P. Delespaul, M. De Hert, C. Derom, E. Thiery, N. Jacobs, M. Wichers, J. van Os, B. P. F. Rutten, S. Gülöksüz, B. Klingenberg

**Affiliations:** ^1^Faculty of Health, Medicine and Life Sciences (Honours Programme); ^2^Psychiatry and Neuropsychology, Maastricht University, Maastricht, Netherlands; ^3^University Psychiatric Centre, KU Leuven; ^4^Centre of Human Genetics, University Hospitals Leuven, Leuven; ^5^Department of Neurology, Ghent University Hospitals, Ghent, Belgium

## Abstract

**Introduction:**

Neuroticism has societal, mental and physical health relevance, with an etiology involving genetic predisposition, psychological influence, and their interaction.

**Objectives:**

To understand whether the association between polygenic risk score for neuroticism (PRS-N) and neuroticism is moderated by affective well-being.

**Methods:**

Data were derived from TwinssCan, a general population twin cohort (age range=15-35 years, 478 monozygotic twins). Self-report questionnaires were used to measure well-being and neuroticism. PRS-N was trained from the Genetics of Personality Consortium (GPC) and United Kingdom Biobank (UKB). Multilevel mixed-effects models were used to test baseline and changes in well-being and neuroticism.

**Results:**

Baseline wellbeing and neuroticism were associated (β=-1.35, p<0.001). PRSs-N were associated with baseline neuroticism (lowest p-value: 0.008 in GPC, 0.01 in UKB). In interaction models (PRS x wellbeing), GPC PRS-N (β=0.38, p=0.04) and UKB PRS-N (β=0.81, p<0.001) had significant interactions.

PRSs-N were associated with changes in neuroticism (lowest p-value: 0.03 in GPC, 0.3 in UKB). Furthermore, changes in wellbeing and neuroticism were associated (β =-0.66, p<0.001). In interaction models (PRS x change in wellbeing), only UKB PRS-N had a significant interaction (β=0.80, p<0.001).

**Conclusions:**

Interaction between polygenic risk, wellbeing and neuroticism, were observed regarding baselines measures and change over time. Depending on the analysis step, the direction of the effect changed.

**Disclosure of Interest:**

None Declared

